# Fixational saccades are more disconjugate in adults than in children

**DOI:** 10.1371/journal.pone.0175295

**Published:** 2017-04-13

**Authors:** Aasef G. Shaikh, Fatema F. Ghasia

**Affiliations:** 1 Department of Neurology, Case Western Reserve University, Cleveland, OH, United States of America; 2 Neurology service, Louis Stokes Cleveland VA medical center, Cleveland, OH, United States of America; 3 Daroff-Del’Osso Ocular Motility Laboratory, Louis Stokes Cleveland VA medical center, Cleveland, OH, United States of America; 4 Cole Eye Institute, Cleveland Clinic, Cleveland, OH, United States of America; State University of New York Downstate Medical Center, UNITED STATES

## Abstract

**Purpose:**

Fixational eye movements are of particular interest for three reasons. They are critical for preventing visual fading and enhancing visual perception; their disconjugacy allows scanning in three dimensions, and their neural correlates span through the cortico-striatal, striato-collicular and brainstem networks. Fixational eye movements are altered in various pediatric ophthalmologic and neurologic disorders. The goal of this study was to compare the dynamics of fixational eye movements in normal children and adults.

**Methods:**

We measured the fixational saccades and inter-saccadic drifts in eye positions using infrared video-oculography in children and adults. We assessed the frequency, amplitude, main-sequence, and disconjugacy of fixational saccades as well as the intra-saccadic drift velocity and variance between these two groups.

**Results:**

We found a similar frequency but an increase in the amplitude of fixational saccades in children compared to adults. We also found that the fixational saccades were more conjugate in children than in adults. The inter-saccadic drifts were comparable between the two groups.

**Discussion:**

This study provides normative values of dynamics of fixational eye movement in children and adults. The greater disconjugacy of fixational saccades in adults suggests the existence of neural mechanisms that can independently regulate the movements of two eyes. The differences between adult and pediatric populations could be due to completion of the development of binocularly independent regulation of fixational saccades nearing adulthood. The alternate possibility is that the increased disconjugacy between the two eyes may represent a deficiency in the eye movement performance as a function of increasing age.

## Introduction

The basic construct of the nervous system takes place early during the development, but its refinement is an ongoing process that lasts through adulthood [1–3]. Study of eye movement development has provided proof of this principle. Smooth tracking of a moving target (ocular pursuit) is immature at birth, but it improves in the first year of life [4–8]. Neural infrastructure to generate saccades is in place at infancy, but saccadic accuracy improves throughout the childhood. Although controversial, saccade velocity appears to be slower in adults than in children [9–15].

The ability to hold the gaze steady is not well developed at birth but is acquired in the first few months of life [16]. Ocular misalignment in newborns through the first two months of life reflects normally developing vergence system[17]. The frequency of such misalignment progressively reduces in infancy with the addition of the retinal disparity cues to the stimulus [18]. Although present since early infancy, the fixation stability continues to improve through adolescence. The frequency of intrusive saccades as well as larger amplitude saccades during fixation decreases through the mid-teenage years, thereby improving the gaze-holding function [15, 19]. Steady gaze is not the only requirement for clear vision. Miniature fixational eye movements such as microsaccades and drifts are critical for clear visual perception and prevention of visual fading [20–26]. They also constitute an effective sampling strategy by which the visual system enhances the processing of spatial detail [27–29] and extracts useful information from a visual scene [30, 31].

Fixational eye movements are affected by various pediatric neurologic disorders like periventricular leukomalacia and cortical visual impairment as well as ophthalmic disorders like strabismus and amblyopia [31–35]. There is a paucity of literature of the dynamics of fixational saccades and inter-saccadic drifts in normal children. One reason for such fundamental investigation is that it addresses major questions centered on the role of microsaccades in the pathophysiology of various disorders of the development of the visual system such as amblyopia. Multiple studies have reported greater fixational instability in amblyopes compared to normals [31, 36, 37]. This instability may be related to altered microsaccade [31, 38], drift [37] or both microsaccades and drift [39]. Also, the greater fixational instability in amblyopic eye compared to fellow eye implies that there is a greater disconjugacy in amblyopes compared to normals. Thus, greater disparities in fixational eye movements may not be beneficial and in fact presence of it in young children could hamper the visual development. Here we set out to investigate the dynamics of fixational saccades and inter-saccadic drifts in normal children and compare them to adults.

## Methods

Eye movements were measured and analyzed from fourteen healthy adults (seven men and seven women, age range 24–36) and ten healthy children (four boys and six girls, age range 5–13). All subjects had comparable best-corrected log MAR visual acuity with no other ocular or neurologic abnormalities (Table 1). Healthy siblings from the author’s pediatric ophthalmology practice were invited to participate in the study. Older family members, hospital or university employees participated as healthy adults. The subjects were recruited betweeen 2014–2016. The subjects did not have any psychiatric or neurological diagnosis, and they were not on psychotropic, nervous system enhancer, or suppressant medications. All the subjects had a comprehensive eye exam including a cycloplegic refraction in children. All participants wore appropriate refractive correction for the experiments. Adult patients with higher refractive errors wore contact lenses for the experiments.

**Table 1:** Demographics and log BCEA of fixation data of all the study participants.

	Category	Age	Refraction OD (SE Diopters)	Refraction OS (SE Diopters)	Log BCEA
1	child	5	+1.5	+1.5	-0.52
2	child	6	+1	+1	-0.23
3	child	6	+1	+1	0.20
4	child	8	+1	+1	-0.95
5	child	9	+0.25	+0.25	-0.64
6	child	10	+0.5	+0.5	-1.09
7	child	10	-2	-2	-0.53
8	child	11	-1.5	-1.5	-0.50
9	child	12	-3	-3	-0.30
10	child	13	-7	-7	-0.16
11	adult	24	-1.25	-1.25	-0.54
12	adult	26	-4.5	-4.5	-0.55
13	adult	27	-7.5	-7.5	-0.40
14	adult	28	-2.5	-2.5	-0.39
15	adult	29	-3.25	-3.5	-0.10
16	adult	30	-4.75	-4.5	-0.56
17	adult	30	-7	-7	-0.38
18	adult	31	-3.25	-3	-0.46
19	adult	31	plano	-1.25	-1.05
20	adult	32	-2.25	-1.5	-0.22
21	adult	32	-3.25	-2.25	-0.75
22	adult	32	-4.5	-4.5	-0.41
23	adult	34	-8	-8	-0.50
24	adult	36	-2.5	-2.5	-0.65

SE: Spherical equivalent, BCEA: bivariate contour elliptical analysis

### Eye movement measurements

High-resolution video-based eye tracker (EyeLink 1000^®^, SR Research, Ontario, Canada) was used to measure binocular horizontal and vertical eye positions. This system has a spatial resolution of 0.01° and a temporal resolution of 500 Hz, which enables the detection of minute differences. This method allows identification of saccadic amplitude of 0.1° and higher. Experiment protocols were approved by the Cleveland Clinic institutional review board and complied with the tenets of the Declaration of Helsinki. All subjects provided written informed consent. The informed consent was obtained from the parents, or legal guardians on behalf of minors/children enrolled in the study.

We used the same experimental protocol in all subjects from both groups. Each subject's head was immobilized using a headrest with forehead and chin support. The headrest was positioned 55 cm away from the LCD screen. The subjects were asked to fix their gaze on a red non-patterned target (0.5° visual angle) that was projected on the LCD screen on a white background in a completely dark room for 45 seconds. The target was presented in the center of the experiment display. The target’s luminance was 250 cd/m^2^. The experiment was conducted in a dark room. The experiment trial began with the calibration and validation per the manufacturer’s specifications. The examination protocol was part of a study measuring our laboratory’s normative data for gaze holding and visual guided saccades in adults and children.

### Data analysis

*Saccade* analysis began with the removal of blinks defined as portions of the raw data where the pupil information was missing. We also removed data where there was a sudden change in the pupil size defined as >50 units/sample, as they would probably be partial blinks where the pupil is not completely occluded. We removed 200 milliseconds of data trace before and after each blink or partial-blink to exclude epochs where the pupil could be partially occluded [20, 40]. Eye position was differentiated to compute velocity. Fixational saccades were identified using two algorithms (1) an unsupervised clustering method [41] and (2) Engbert and Kliegl algorithm [42, 43]. The manuscript outlines the results from the unsupervised clustering technique whereas the results from Engbert-Kliegl algorithm are outlined in the supplementary section.

Fixational saccade rate was computed as the number of events in one minute. Small rapid eye movements in the opposite direction called dynamic overshoot followed some saccades. We identified dynamic overshoot by their latency shorter than 20 ms between two movements and did not consider them as a “new” saccade. Thus, the saccade amplitude was defined as the absolute difference between the eye positions at start and end of the saccade. We separately considered horizontal and vertical saccade amplitude, as well as their vector sum of components along both axes (here called “amplitude”) for further analysis.

We also quantified the fixation stability by measuring a bivariate contour ellipse (BCEA) [44]. This method quantifies variability in eye position over a given percentage of the time, in our analysis 68.2%. It also takes into account the correlation between horizontal and vertical positions and has been used previously to measure fixation stability in clinical vision research[36, 37, 44]. The calculation was done using the following equation

$$BCEA=\pi \chi^{2}\sigma_{x}\sigma_{y}\sqrt{1-p2}$$

σ _x_ σ_y_ are the standard deviation of eye position in the horizontal and vertical meridian respectively, *p* is the product moment correlation of the two position components, and Χ ^2^ is a chi-square variable with two degrees of freedom. A log_10_ transformation was used to normalize the resulting BCEAs. Lower BCEA values indicate more stable fixation.

*Drifts* were defined as epochs between fixational saccades and blinks. We removed 20 msec data at the beginning and end of each of the drifts o to exclude periods of acceleration and deceleration of the eye during fixational saccades and blinks. The vector sum of horizontal and vertical eye velocity (here called “eye velocity”) and the variability of eye position of the ocular drifts was computed. To measure eye velocity, we differentiated eye position signal using Matlab^TM^ (Mathworks, Natick, MA) diff function. Differential value (velocity signal) was further smoothened with Savitzkey-Golay filter, a function that can be applied to a set of digital data points for the smoothing purpose. This technique does not distort the parent data, and we have used a similar approach in our previous paper on fixational movements in amblyopic children[31].

The Kolmogorov-Smirnov test and non-parametric Mann-Whitney tests were used to compare the distribution of fixational saccade amplitude in both groups. Spearman’s rank correlation coefficient was computed to assess the presence of correlation between movements of the right and left eye during microsaccade or drift. Non-parametric Mann-Whitney tests were further utilized to compare distributions of the correlation coefficient measured from each subject.

## Results

**Fig 1:**
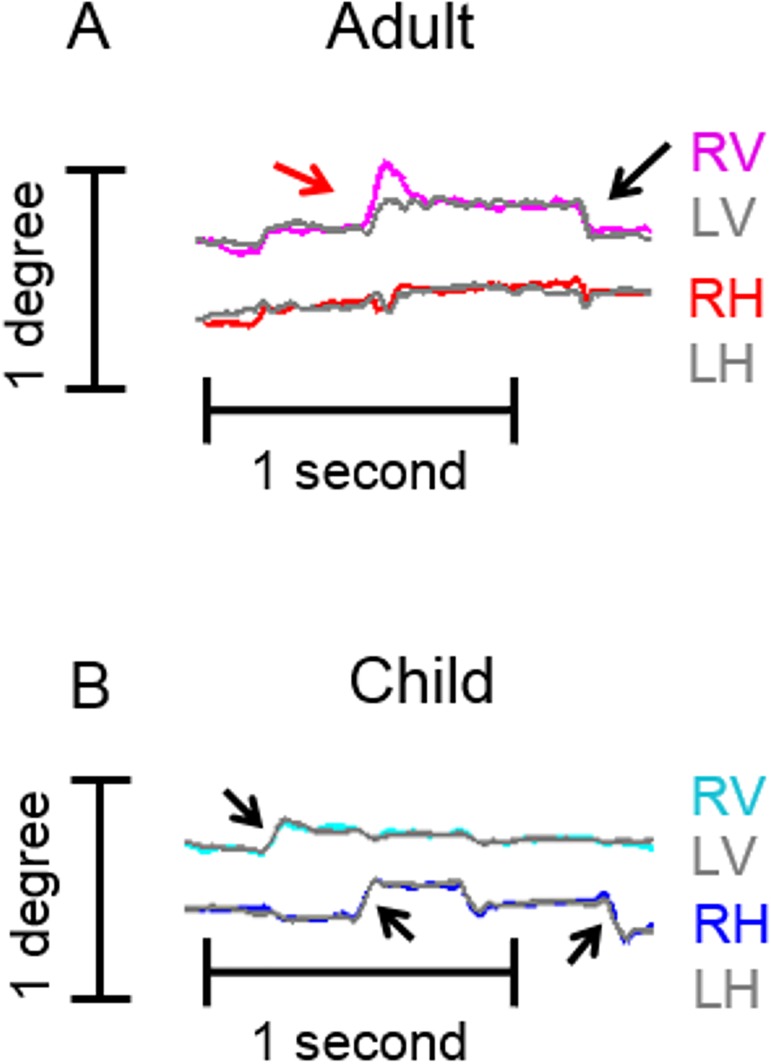
Example of fixational saccade and drifts in a 1.5-second epoch of eye positions recorded from an adult and a child. Horizontal and vertical eye positions were recorded from the right and left eye (LV: left vertical; RV: right vertical; LH: left horizontal; RH: right horizontal). Eye positions are plotted on y-axis while x-axis depicts the corresponding time. Colored traces depict right eye position while gray traces illustrate the left eye. Arrows depict fixational saccades. (A) An example of fixational eye movements in an adult subject (subject 20 in Table 1). The fixational saccade has different amplitudes in the vertical direction (red arrow) between the two eyes suggesting a disconjugacy. (B) An example of fixational eye movement in a child (subject 7 in Table 1). Arrows depict fixational saccade. The amplitude of the fixational saccade is equal suggesting their conjugate nature in the child.

Fig 1 illustrates a 1.5-second epoch of eye position when one adult (Fig 1A) and one child (Fig 1B) held their gaze on a target. The traces show the miniature saccades or fixational saccades (arrows) and the intervening slow drifts in the eye position. The fixational saccades were more disonjugate in the adult subject as evidenced by an amplitude mismatch.

**Fig 2:**
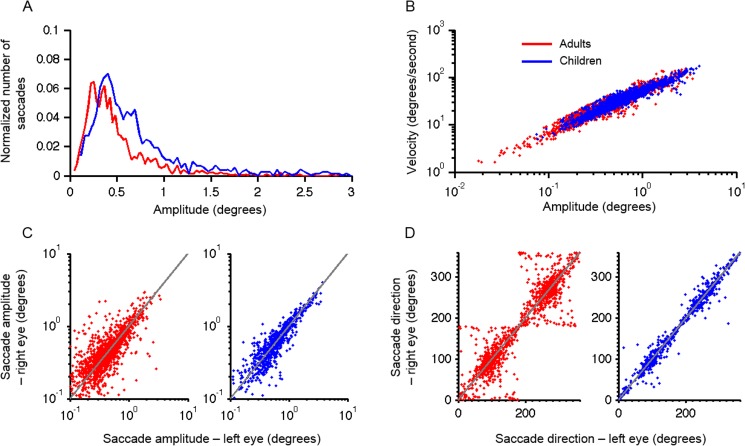
(A) Distribution of microsaccade amplitude, when saccades were identified using clustering algorithm proposed by Otero-Millan et al. (2014). A normalized number of microsaccades are plotted on the y-axis, while x-axis depicts the amplitude of microsaccades in degrees. Red lines depict the distribution of microsaccades in adults, while blue lines depict children. The two distributions were significantly different (Two-sample Kolmogorov-Smirnov test p<0.0001). (B) Kinematic properties of microsaccades quantified in the main-sequence analysis. Eye velocities are plotted on the y-axis while the corresponding positions are plotted on the x-axis. Each data point depicts one saccade. Blue symbols illustrate children, while red data points are adults. (C) Comparison of the amplitude disconjugacy and (D) directional disconjugacy of fixational saccades. In both panels, the right eye is plotted on y-axis while the left eye is plotted on the x-axis. Red symbols depict adults, while children are shown in blue data points. Grey line is an equality line. The red points, suggesting adults, have larger scatter showing more amplitude and directional disconjugacy compared to pediatric patients.

Fig 2 depicts the summary of the quantitative analysis of fixational saccades identified using the clustering algorithm [41]. The histograms summarize the amplitude distribution (Fig 2A) of the children and adult populations. The histograms were normalized by dividing the value of each bin by the peak of the cumulative sum value. There was a significant difference between two distributions with greater amplitude in children compared to adults (Two-sample Kolmogorov-Smirnov test p<0.0001). The median fixational saccade amplitude in adults was 0.4 degrees (25 and 75 percentile: 0.26–0.62 degrees); while in children, it was 0.55 degrees (25 and 75 percentile: 0.36–0.84 degrees). Mann-Whitney U test comparing two samples revealed similar findings. Mean ± standard deviation amplitude in adults was 0.49 ± 0.37, while it was 0.71 ± 0.56 in children (p < 0.0001 Mann-Whitney U test).

We assessed the kinematics of fixational saccades by comparing their amplitude with the peak velocity in the main-sequence analysis. Such a relationship follows the systematic trend between the amplitude and the velocity. Fig 2B illustrates the main-sequence where the scatter of blue data points depicting the summary of 10 children and red symbols representing 14 adults superimpose. The mean slope of the fitted linear function though the data set from each subject was measured. The slope of the linear fit was 44.9 ± 4.2 in adults and 44.6 ± 5.1 in children. The difference was not significant (t-test, p = 0.4).

The subsequent analysis compared the disparity of fixational saccade amplitude in the right (y-axis) versus left eye (x-axis) in both age groups (Fig 2C). Each data point depicted a single fixational saccade. Precisely conjugate fixational saccade would fall on the equality line while the lack of correlation in the scatters from the equality line quantifies the disparity in the amplitude of microsaccades. We sampled between 70–90 microsaccades from each subject. Hence we could measure the correlation coefficient of the disparity between right versus left eye microsaccade amplitude in all subjects. We found that adults had a larger dispersion suggesting more disparity between the two eyes. The value of correlation coefficients was smaller (0.52 ± 0.25) in adults compared to children (0.82 ± 0.15). The difference was statistically significant (p = 0.003, Mann-Whitney U test).

We then compared the disparity in the trajectories of the fixational saccades in the right (x-axis) versus the left (y-axis) eye in both age groups (Fig 2D). We computed a correlation coefficient of the amount of dispersion of the trajectory of the right versus left saccade direction in all subjects. The mean ± standard deviation value of the correlation coefficient measured from all subjects was 0.86 ± 0.14 in adults and 0.95 ± 0.05 in children; the difference was significant (Mann-Whitney U test, p = 0.01).

S1 Fig summarizes the quantitative analysis of fixational saccades identified using Engbert and Kliegl algorithm [42, 43]. The results re-demonstrated the differences in the distribution of the amplitude of fixational saccades in adults compared to the children (panel A). The mean (± standard deviation) amplitude in adults was 0.46 ± 0.38, whereas it was 0.62 ± 0.52 in children, the difference was statistically significant (p<0.0001, Mann- Whitney U test). In subsequent analysis, we assessed whether fixational saccades identified with Engbert and Kliegl algorithm depict differences in amplitude disconjugacy in adults compared to children. The comparison of the amplitude disconjugacy of the values of fixational saccade amplitude in both age groups is depicted in panel B. Larger scatter suggested more disconjugacy. The comparison of the directions of the fixational saccade trajectories re-demonstrated larger directional disconjugacy in adults compared to children (panel C). In order to quantitatively assess the right-left directional difference, we individually measured the correlation coefficient assessing the dispersion for each subject. The difference in population of correlation coefficients was statistically significant (Mean correlation coefficient adult: 0.63 ± 0.13, while it was 0.86 ± 0.03 in children; Mann-Whitney U test, p<0.0001).

The previous analysis focused on the vector summation of horizontal and vertical components of the fixational saccades. Here we asked whether the disparity in saccade amplitude seen in adults is mainly representative of disconjugate horizontal or vertical component. Therefore we independently analyzed the correlation coefficient depicting the dispersion of the amplitudes of right versus left eye horizontal components and right versus left eye vertical components of the microsaccades. Mean (± standard deviation) of the correlation coefficients measured from the adults were 0.67 ± 0.28 and 0.53 ± 0.34 for horizontal and vertical saccades respectively. In children, these values were 0.87 ± 0.06 and 0.73 ± 0.34 for horizontal and vertical saccades respectively. Both horizontal and vertical components of fixational saccades were more disconjugate in adults than in children (p<0.05 Mann-Whitney U test). A smaller value of correlation coefficient suggests a larger disconjugacy in vertical than horizontal components of the fixational saccades in both adults and children (p<0.05 Mann-Whitney U test).

We also measured the direction of the right and left eye movements elicited during a fixational saccade and calculated the percentage of saccades that were in the same or opposite direction. In adults, the vertical saccade directional disparity was greater (28.2 ± 8.2%) with a larger number of microsaccades elicited in the opposite directions compared to children (11.9 ± 4.8%) (p <0.0001- Mann-Whitney U test). On the other hand, the amount of horizontal saccade directional disparity were comparable for both adults (8.0 ± 7.6%) and children (7.8 ± 4.6%) (p = 0.48- Mann-Whitney U test).

The rate of microsaccades was measured in both groups; the mean rate was 108.9 ± 44.3 per minute in adults and 105.1 ± 20.9 per minute in children, the difference in mean was not significant (t-test; p = 0.8).

In the subsequent analysis, we individually measured the drift velocity of the right and the left eye. The median drift velocity was 2.82 degrees/second, and 95% confidence interval was 2.8–2.9 degrees/second in adults. In children, the median drift velocity was 2.76 degrees/second and 95% confidence interval was 2.6–2.8 degrees/second. Drift velocities in adults and children were not significantly different (Two-sample Kolmogorov-Smirnov test; p = 0.44; k = 0.12). We then assessed whether there were differences in the drift velocity between the right and the left eye at a given time. The differences would be evident in the form of dispersions from the line of equality correlating the right versus left eye drift velocity. There was a large dispersion with a weak correlation between the drift velocity of the right versus left eye. The correlation was weak in both groups (adults: 0.18 ± 0.17 children: 0.1 ± 0.08, Mann-Whitney U test, p > 0.1).

We also measured the bivariate contour ellipse analysis of children and adults (Table 1). The mean log BCEA of children (-0.47 ± 0.37) was comparable to adults (-0.49 ± 0.22), Mann-Whitney U test p = 0.7.

## Discussion

The main findings of the current study analyzing the fixational eye movement characteristics in healthy children and adults are as follows: 1) fixational saccades in adults and children had similar frequencies with children having a greater amplitude of fixational saccades than adults. 2) fixational saccades were more disconjugate in adults than in children. 3) adults and children had disconjugate drifts with comparable drift velocity.

Binocular single vision is achieved due to a combination of vergence and sensory fusion. Binocular coordination during visual fixation is critical as it is a prerequisite for fusion. Panum’s area is defined as the maximum amount of disparity or misalignment between the two eyes that can be fused into a single percept. The classical account of Panum’s area is a static zone of fixed extent. Panum’s fusional area is elliptical with sensory fusion possible over a larger range of horizontal disparities compared to vertical disparities [45] [46]. The size of the horizontal Panum’s fusional area is about 20 arc min whereas the vertical Panum’s fusional area is 8 arc min at low spatial and temporal frequencies. A slight disparity between the images of the two eyes could be beneficial as it facilitates 3D vision and stereopsis. However, disparities of more than 0.25 deg can result in diplopia with degradation of stereopsis [45, 47]. We found that fixational saccades were most disconjugate in the vertical direction in adults. This finding was surprising, as we would have expected to see a greater disparity in the horizontal meridian than the vertical meridian given the elliptical nature of the Pannum’s fusional area. Nonetheless, both horizontal ((0.13 ± 0.20; 0.12 ± 0.13) and vertical disconjugacy (0.16 ± 0.23; 0.11 ± 0.11) values in adults and children respectively fell within the extent of Panum’s fusional area. In other words, the disparity in fixational saccade amplitude did not interfere with the subjects’ ability to fuse the images and achieve stereopsis. We also found greater disconjugacy in inter-saccadic drifts compared to fixational saccades. However, we did not find significant differences in the disconjugacy of drifts between the two groups. Thus, these results disagree with the possibility that more disconjugate microsaccades in adults are secondary to disconjugate drifts.

Larger disconjugacy in vertical compared to horizontal trajectories of microsaccades in adults and children suggests independent control of vertical and horizontal trajectories of the microsaccades. The neural activity recorded from the saccadic burst neurons strongly correlated with the ipsilateral saccade, but not with the movement of the contralateral eye suggesting an independent control of fixational eye movements. Up to 7–8% of microsaccades in monkeys have opposite directions [48]. We found a greater percentage of oppositely0020directed microsaccades between the right and the left eye in adults compared to children. Therefore, it is possible that in addition to the burst, an additional mechanism influences the contralateral eye movement. Such putatively independent mechanism induces disconjugacy and allows vergence.

The fundamental mechanism for microsaccade generation remains the same as for the larger sized visually guided or reflexive saccades [25, 48–55]. Recent physiological and computational studies have suggested a role of collicular stochastic activity for the generation of microsaccades [53]. It is proposed that a simple circuit involving the omnipause neurons and the long lead burst neurons is responsible for triggering the microsaccades. Both these group of neurons receive direct projections from the superior colliculus. The balance of reciprocal innervation between the omnipause neurons and long lead burst neurons modulated by the projections from the superior colliculus triggers the microsaccades. [53, 54, 56, 57]. Using two independent quantitative algorithms[41–43], we found that fixational saccades in adults and children had similar frequencies suggesting equally efficacious burst generation in adults and children as young as five. Our results highlight the lack of difference in the rate of fixational saccades in adults and children, measured using both algorithms. This further confirms early maturation of the circuit that is involved in their generation. We found an increase in the amplitude of fixational saccades in children compared to adults. This is in agreement with previous studies evaluating fixational stability in children. The increased amplitude is thought to be due to an increase in saccadic intrusions [15, 19] versus due to relative inattention[58] in children.

A limitation of the current study is the use of video tracker to record miniature eye movements rather than the scleral search coils, which is a gold standard technique to measure 3D eye movements. Since our subjects were children; use of scleral search coil that requires wearing a coil embedded in a contact lens was deemed difficult. Thus, we opted to use the Eyelink^TM^ video-tracker, which has been extensively used in multiple laboratories to measure fixational eye movements in normal controls [55, 59, 60] and disease states [31, 38, 51, 61]. Occasional studies have questioned the validity of video-based eye trackers in measuring monocular microsaccades [62]. We do not doubt the validity of the video-based tracker in identifying monocular microsaccades and especially the interpretation of our results for two reasons: (1) We used the same technique in adults and children to measure fixational saccades and intra-saccadic drifts and found age-related differences in fixational saccades. These changes are unlikely to be related to the different ocular geometry and the associated anatomical disparity between adults and children as if the later were the case we would have seen a difference in both the fixational saccades and intra-saccadic drifts. (2) If identified saccades were “noise,” then they would not follow the main-sequence. In contrast, as expected all of the eye movements measured by video-oculography followed the main-sequence for saccade. Other limitations include the use of corrective lenses by some of our subjects during the experiment. It is possible that the presence of corrective lenses could alter the amplitude of the detected fixational saccade. Contact lenses have lower RMS with higher saccadic precision. All participants (except one child) with refractive errors > -3 diopters wore contact lenses for the eye movement recording sessions. The results were not different. We also excluded participants with a difference in refractive errors > one diopter between the two eyes or with astigmatism > 0.75 diopters. Thus, any effects of refractive error correction on the disconjugacy of fixational saccades between the two eyes are unlikely. We also asked whether the results between adults and children are due to differing amounts of vergence responses at the testing distance of 55 cm. This possibility is less likely for two reasons. One, we did not find difference in the drifts, if the vergence angle was responsible then we would find differences in both drifts and saccades. Two, previous studies have reported similar amounts of vergence at the viewing distance used in the current study[63–65].

To summarize, we found larger amplitude but less disconjugate fixational saccades in children than adults. The lack of disconjugacy might suggest delayed maturation of the central process that independently regulates fixational saccades of two eyes. The alternate possibility is the increased disconjugacy between the two eyes may represent a deficiency in the eye movement performance as a function of increasing age.

## Supporting information

S1 Fig (A) Distribution of microsaccade amplitude, when saccades were identified using Engbert and Kliegl algorithm[42, 43]. A normalized number of microsaccades are plotted on the y-axis, while x-axis depicts the amplitude of microsaccades in degrees. Red lines depict the distribution of microsaccades in adults, while blue lines depict children. The two distributions were significantly different (Two-sample Kolmogorov-Smirnov test p<0.0001). (B) Comparison of the amplitude disconjugacy and (C) directional disconjugacy of fixational saccades. In both panels, the right eye is plotted on y-axis while the left eye is plotted on the x-axis. Red symbols depict adults, while children are shown in blue data points. Grey line is an equality line. The red points, suggesting adults, have larger scatter showing more amplitude and directional disconjugacy compared to pediatric patients.
(TIF)
