# Supplementary figures and images for: Fixational saccades are more disconjugate in adults than in children
Source: PLoS One. 2017 Apr 13;12(4):e0175295. doi: 10.1371/journal.pone.0175295 (PMC5391133; doi:10.1371/journal.pone.0175295)

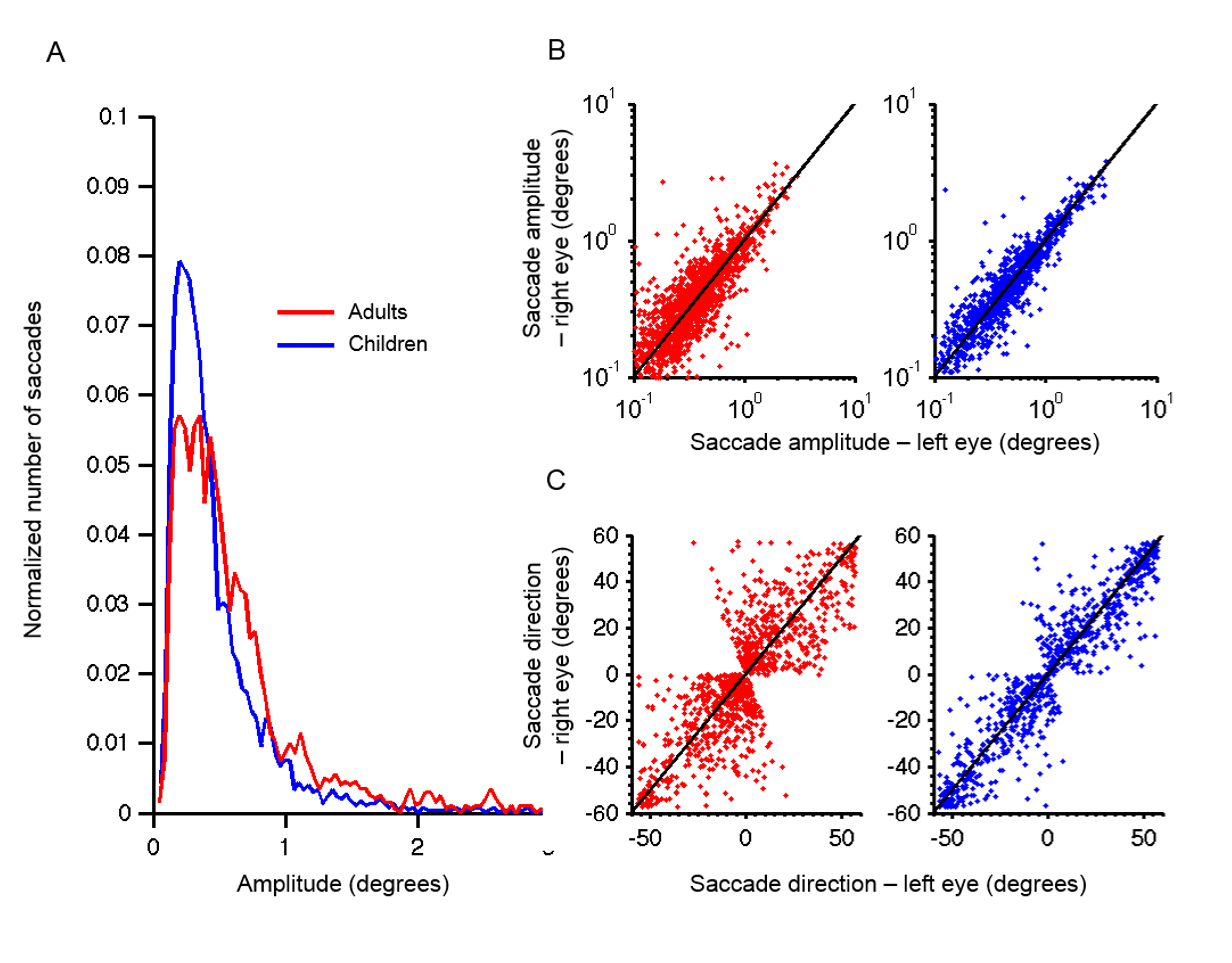

Supplement: S1 Fig — (A) Distribution of microsaccade amplitude, when saccades were identified using Engbert and Kliegl algorithm[42, 43]. A normalized number of microsaccades are plotted on the y-axis, while x-axis depicts the amplitude of microsaccades in degrees. Red lines depict the distribution of microsaccades in adults, while blue lines depict children. The two distributions were significantly different (Two-sample Kolmogorov-Smirnov test p<0.0001). (B) Comparison of the amplitude disconjugacy and (C) directional disconjugacy of fixational saccades. In both panels, the right eye is plotted on y-axis while the left eye is plotted on the x-axis. Red symbols depict adults, while children are shown in blue data points. Grey line is an equality line. The red points, suggesting adults, have larger scatter showing more amplitude and directional disconjugacy compared to pediatric patients. (TIF) [file pone.0175295.s001.tif]
